# Supplementary figures and images for: Amplicon sequencing of pasteurized retail dairy enables genomic surveillance of H5N1 avian influenza virus in United States cattle
Source: PLoS One. 2025 Jun 13;20(6):e0325203. doi: 10.1371/journal.pone.0325203 (PMC12165699; doi:10.1371/journal.pone.0325203)

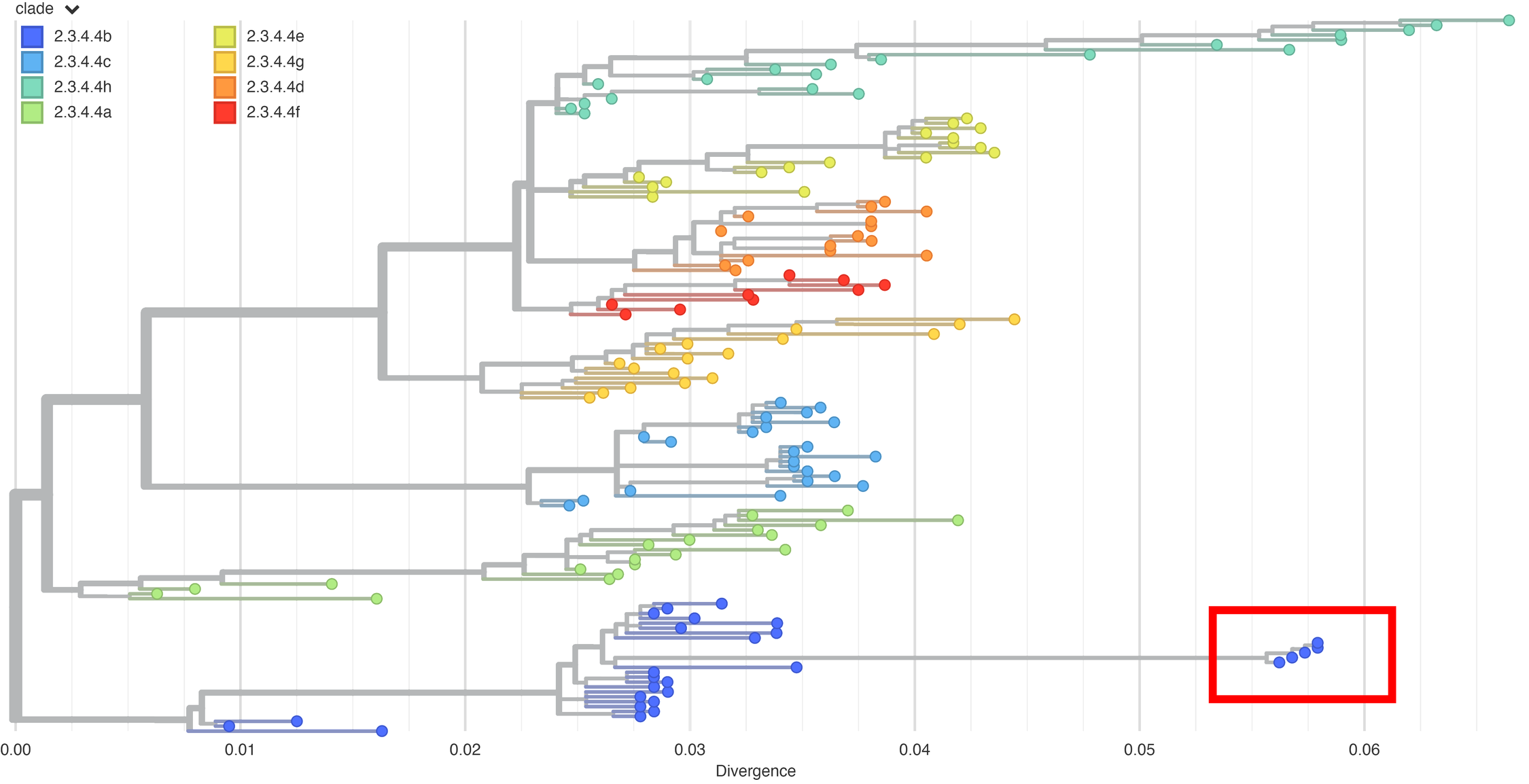

Supplement: S1 Fig — The sequences from this study are in the red box on the lower right side of the chart, branching out from the 2.3.4.4b subclade. (TIF) [file pone.0325203.s001.tif]

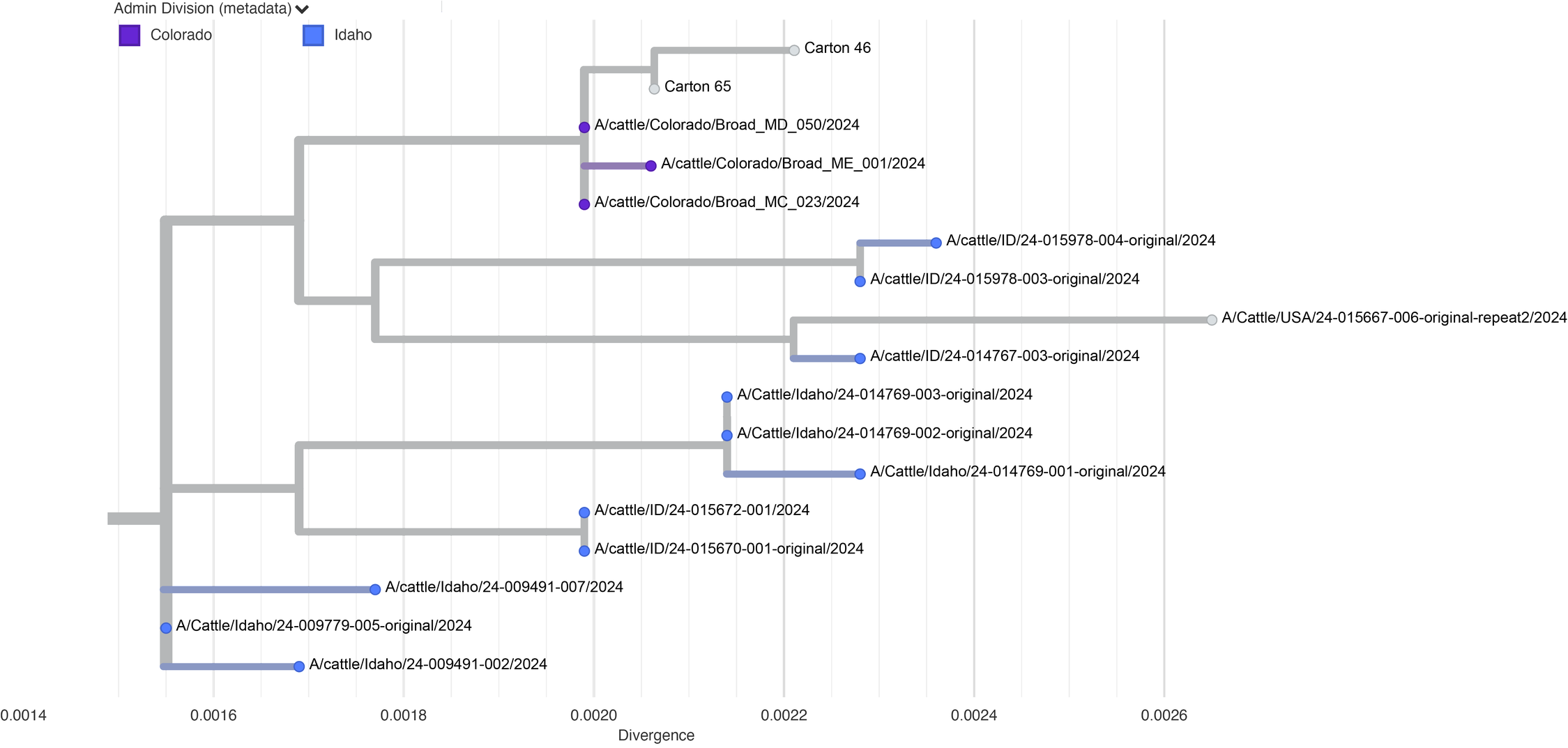

Supplement: S2 Fig — (TIF) [file pone.0325203.s002.tif]

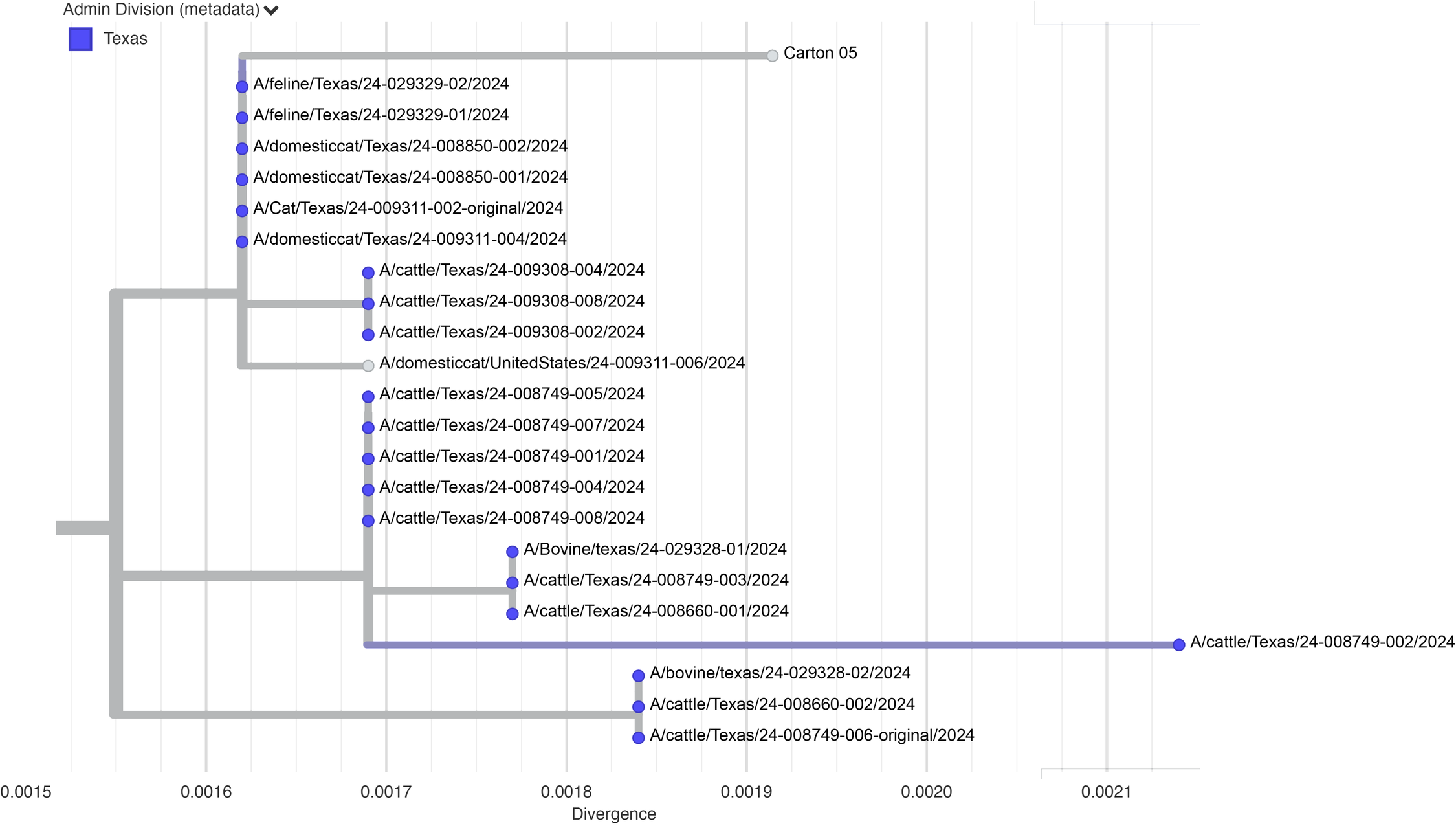

Supplement: S3 Fig — Carton 05 is from Colorado. It clusters locally with feline and bovine sequences from Texas. (TIF) [file pone.0325203.s003.tif]

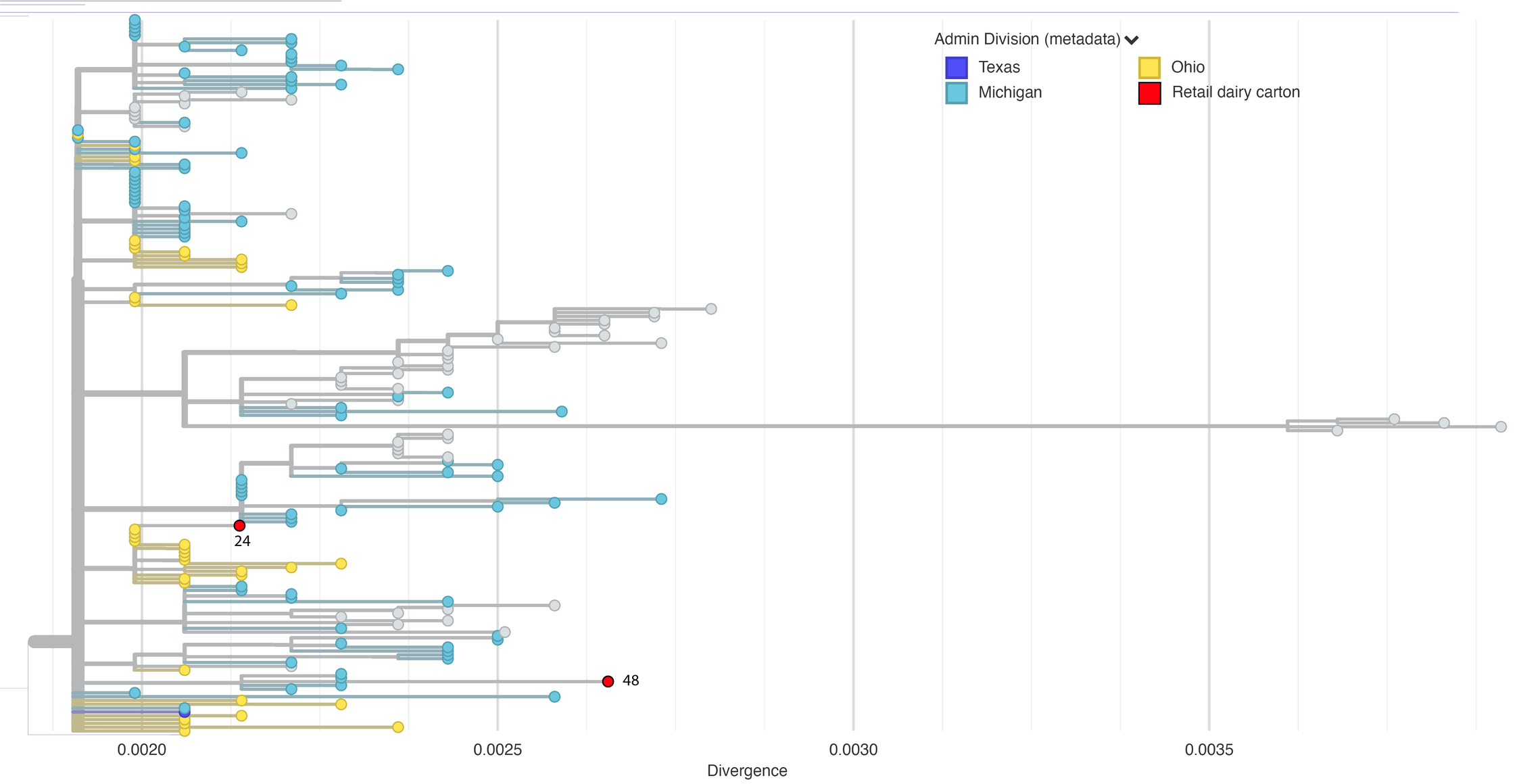

Supplement: S4 Fig — Cartons 24 and 48 (red circles) cluster within a clade of mostly Michigan and Ohio sequences. (TIF) [file pone.0325203.s004.tif]
