# Supplementary material for: Amplicon sequencing of pasteurized retail dairy enables genomic surveillance of H5N1 avian influenza virus in United States cattle
Source: PLoS One. 2025 Jun 13;20(6):e0325203. doi: 10.1371/journal.pone.0325203 (PMC12165699; doi:10.1371/journal.pone.0325203)
Supplement: S1 File — (PDF) [file pone.0325203.s010.pdf]

S1 File:

To test primer specificity in silico, several H5 HA sequences from birds [OR858836.1 (goose, NY 2023), OR819057.1 (peregrine falcon, NY 2022), OQ968076.1 (goose, WY 2022), OQ968052.1 (goose, MN 2022), OQ968012.1 (chicken, WI 2022)] were downloaded and the two sets of selected primers and probes were aligned to ensure they would bind to other 2.3.4.4b sequences from the current outbreak in North American birds. This was done to confirm the primers would continue working in the event of a new introduction from birds to cows. Sequences from A/Victoria/4897/2022 H1N1 and A/Darwin/9/2021 H3N2, two isolates that have been recommended as vaccine components in recent years, were also downloaded to ensure the selected primers and probes would not align to commonly-circulating sequences (https://www.who.int/publications/m/item/recommended-composition-of-influenza-virus-vaccines-for-use-in-the-2024-2025-northern-hemisphere-influenza-season.) The primer sequences did not align to sequences from influenza B viruses B/Austria/13594/7/2021 (B/Victoria lineage) and B/Phuket/3073/2013 (B/Yamagata lineage). To test this specificity in vitro, the two primer/probe sets were tested on H5N1 clade 2.3.4.4b vRNA, A/California/04/2009(H1N1) vRNA, and Influenza A/Kawasaki/173/2001 M gene RNA transcript. Only one set proved specific to the H5 sequence, with the primer sequence 5'-GGGAAGCTATGCGACCTAAAT-3' (forward) and 5'-CATTCCGGCACTCTGATGAA-3' (reverse) and the probe sequence 5'-ACATTGGGTTTCCGAGGAGCCATC-3' with a FAM fluorophore. This set was used for all further analysis.
