# Supplementary material for: Amplicon sequencing of pasteurized retail dairy enables genomic surveillance of H5N1 avian influenza virus in United States cattle
Source: PLoS One. 2025 Jun 13;20(6):e0325203. doi: 10.1371/journal.pone.0325203 (PMC12165699; doi:10.1371/journal.pone.0325203)
Supplement: Attachment — Submitted filename: response_to_reviewers.docx [file pone.0325203.s012.docx]

Dear Dr. Ayi Vandi Kwaghe,

We would like to thank the editor and reviewers for their helpful comments, which have strengthened the manuscript and allowed us to make it more clear. We are excited to send you the revised manuscript, titled "Pasteurized retail dairy enables genomic surveillance of H5N1 avian influenza virus in United States cattle." We hope that it is now acceptable for publication in *PLOS ONE*.

## Journal Requirements:

*Please ensure that your manuscript meets PLOS ONE's style requirements, including those for file naming.*

We have reformatted the manuscript to match the requirements.

*Thank you for stating the following in the Competing Interests section:*

*“I have read the journal's policy and the authors of this manuscript have the following competing interests: AJL has received travel funding from Oxford Nanopore Technologies to present this work at a conference.”*

*Please confirm that this does not alter your adherence to all PLOS ONE policies on sharing data and materials, by including the following statement: "This does not alter our adherence to PLOS ONE policies on sharing data and materials.”*

AJL has received travel funding from Oxford Nanopore Technologies to present this work at a conference. This does not alter our adherence to PLOS ONE policies on sharing data and materials.

*Please include captions for your Supporting Information files at the end of your manuscript, and update any in-text citations to match accordingly.*

These captions have now been added.

*Please review your reference list to ensure that it is complete and correct... Any changes to the reference list should be mentioned in the rebuttal letter that accompanies your revised manuscript.*

We have updated several references. Preprints by Wallace et al. [1], Hu et al. [2], and Spackman et al. [3] have been published in journals, and their references have been updated to reflect this. Reviewer #6 pointed us towards the need to cite Caserta et al. [4] The same reviewer also suggested we perform a Spearman correlation, and we cited the tool we used for this [5]. A comment from reviewer #2 revealed that we had omitted citations to Rehman et al. [6] and the CDC's H5N1 Genetic Changes Inventory [7]. Those citations are now included in the manuscript. Additionally, we updated our citation of the USDA's plans for nationwide milk testing now that those policies are formalized [8].

## Reviewers' Comments:

### Reviewer #1:

*It is not clear from the data presented what the sensitivity and specificity of the technique might be. This information is needed to assess the value of the protocol from a diagnostic perspective.*

We agree that a rigorous treatment of sensitivity and specificity would be beneficial, but it must be noted that this is not a diagnostic protocol. We cannot recommend using this method for clinical samples. We perform RT-qPCR as a way to prioritize samples to sequence. If we need a diagnostic qPCR for a sample, we reach out to colleagues at the Wisconsin Veterinary Diagnostic Lab, where they have systems in place to perform diagnostic assays. Anecdotally, every sample we have sent for confirmation with the WVDL has been confirmed, so we are confident that our assay is not producing high numbers of false positives.

As far as the sensitivity of the sequencing method itself, the lowest copies/mL sample we were able to sequence well had 291 copies/mL of dairy product. We continue trying to sequence samples with lower amounts of RNA to find the lower limit of our sequencing approach.

*Please clarify the statement '....Human infections have also been observed in poultry workers after presumed spillover of HPAI into poultry from cattle, underscoring the zoonotic potential of this outbreak (9).- currently it does not make sense and is not supported by the reference* [*https://www.cdc.gov/mmwr/volumes/73/wr/mm7334a1.htm*](https://urldefense.com/v3/__https://www.cdc.gov/mmwr/volumes/73/wr/mm7334a1.htm__;!!Mak6IKo!OaACwpYYR_y-vymeqqDyO3IGwSpLiDM7HpS92crmgcyQg4kTyOE5ZRDW0C2uuBfpan25iA6R97Aximw$)*.*

We have changed the wording of the sentence to "Poultry workers have also been infected, underscoring the ability of this virus to cross species barriers." (line 55)

*As presented, this study does not fully align with a 'One Health approach' which is usually considered to be a transdisciplinary integrated approach to understanding a complex problem vs a focused study on a technique applied to detecting a potential pathogen which is zoonotic.*

We appreciate this feedback and we agree that this study is only a start towards a true One Health approach. However, our approach is designed to monitor animal health through retail food products, which draws a clear connection to the One Health concept that human health is connected to environmental and zoonotic factors. In developing our workflow, we worked with non-academic regulators on the human and animal health fronts to understand the full scope of this complex problem.

*There is mention in the conclusions about further sampling of pig farms to detect AIVs but no real consideration of how such data could be used in epidemiological studies and risk assessments. Can this be further discussed ?*

We have added to the discussion (lines 445-455) to include further emphasis on these points.

### Reviewer #2:

*There seem to be discrepancies between numbers in the text and tables. While it is clear that the authors have tested 66 cartons in 2 months, positives are described as coming from 5 states in the abstract/text but seem to come from just 3 in Table 2. While 18 cartons are positive according to table S3, 10 are positive in Table 2 and 13 described as positive in the text. Please clarify. The Ct value indicated for carton 28 (68.48) also seems off.*

Well noted. There were 66 cartons tested, and 13 of those were positive. Those 13 included samples from 5 different states. We apologize for the confusion caused by Table S3, which was taken directly from our github repository. Edits have been made to remove redundancy in instances where we tested the same carton multiple times, and to place samples that were tested in duplicate via different assays near each other. There are 13 positive cartons as tested by RT-qPCR. Three of the samples (cartons 19, 27, and 41) failed pre-sequencing quality control and sequenced poorly during our method development. We now specifically name those samples in the text (line 265). Unfortunately, these three samples accounted for two of the states with positives. Table 2 includes the 10 samples (from 3 states) that we attempted to sequence in this study.

The erroneous Ct value should not have been included in the table. This sample was inconclusive, with one positive replicate and the other negative. We have decided to not report inconclusive samples, so it has been removed.

*How did you set your thresholds? Both for the 20x sequence coverage chosen and for the 10% frequency for the variants search.*

For the 20% coverage threshold, we wanted to make sure that we chose a threshold that was well above the coverage we saw in the negative controls. While a large majority of the negative controls had less than 10x coverage, there were a few regions that exceeded 10x. None of the coverage ever exceeded 20x in our negative controls, which gives us confidence that any coverage above that threshold is real and meaningful data from our samples.

For the variant frequency threshold, we wanted to have a frequency that reflected multiple reads. We did not want to report variants as being present if there was only one read that supported that variant. Assuming the coverage is the lowest it can be (20x) two reads would be 10% frequency. While it is likely that many positions are covered more deeply than the minimum, we still felt like a 10% cutoff allows us to analyze low-frequency variants without including spurious signals.

*Mammalian adaptation markers are known. It is a pity that the authors did not screen their sequence data for those (irrespective of the variants frequency actually, it would be quite informative in a One Health perspective as stressed in the Introduction and Discussion sections).*

Thank you for pointing out this omission. We did compare our observed mutations to a list of known adaptation and virulence markers in influenza, but we should have reported the lists of virulence markers we used. We used tables presented in Nguyen et al., Rehman et al., and the CDC's H5N1 Genetic Changes Inventory. These citations are now added to the references for this manuscript. We reported the results of the screening in the results section under "Few major amino acid substitutions were detected" (lines 336-340). We chose to avoid replicating those previous publication's lists when we only detected a handful of variants.

*Figure 2: the tree is not very informative. What do divergence percentages refer to? What is the scale? The Figures legends are very short and would deserve more info.*

We have expanded on the figure legends to address these concerns. In Figure 1, we added an explanation about the coverage drop-off at certain amplicons. For Figure 2, we have explained the divergence metric and mentioned that gray points have missing metadata.

### Reviewer #3:

*Authors may improve the discussion section by discussing the global implications of the findings and how the approach could be adapted for use in other regions or for monitoring other zoonotic pathogens.*

We have added this sentence in the discussion: "In fact, sampling retail milk to detect cattle outbreaks could be implemented in any area where the production region of milk is available at the point of sale, not just in Canada and the US." (lines 442-444)

We also added more detail into our discussion section addressing swine flu monitoring (lines 445-455), but the pros and cons of our methods in that discussion are broadly applicable to any novel implementation of this approach.

*Also, they have to clearly articulate the limitations of using retail dairy for surveillance, such as sample representativeness, phylogenetic challenges, and potential for false negatives.*

We addressed sample representativeness on line 383, writing "This reduces the effectiveness of retail milk sampling for surveillance purposes because some farms and dairy processors produce little or no product that could be sampled as retail fluid milk." Addressing phylogenetic challenges, we write on line 396: "This limits the utility of phylogenetic analyses, which assume that each sequence represents a single infected individual." With respect to the potential for false negatives, we have added a few sentences on lines 391-394 explaining this further - "While a sample that tests positive from an area is an indicator of H5N1 infection, the reverse is not true. There are many reasons why H5N1 infections may not be picked up by this sampling strategy, so a negative test from a given area is not a declaration that influenza is absent in that area." We hope these edits address these concerns.

### Reviewer #4:

Reviewer #4 left no points to address.

### Reviewer #5:

*1. Paragraph 6, you don’t need to mention “the home state of the researchers on this study”, instead you might need to give more other interesting point about Wisconsin besides “the second- largest dairy producing state in the US”.*

We have removed the reference to Wisconsin as the home state of the researchers.

*2. It is better to put a reference about the existing workflow from your work in SARS-CoV-2 sequencing, as you mentioned it in introduction.*

We now refer to QIAseq Direct on line 88 in the introduction.

*3. It is better to give reference to “OHHLEP’s 4 Cs” strategy which you also mentioned it in introduction.*

We have added a reference to OHHLEP's 4 Cs in the introduction on line 91.

*4. You mentioned in “sample collections” about the dairy products which you took as sample, can you explain about the percentage of each sample taken? How many percent of whole milk did you take and how many percent low-fat wilk did you take? Etc.*

We aimed to sample whole milk (4% cream) if it was available. Approximately 50% of our samples were whole milk, while increasingly small numbers were cream (7 samples), skim milk (7 samples), lowfat milk (5 samples), and half and half (4 samples). We got either one or two samples of yogurt, chocolate milk, kefir, and buttermilk.

*5. It is unclear about how you finally only mentioned about samples from 10 sample cartons from 66 samples which you previously mentioned.*

See previous response to reviewer #2's question about the same subject. We hope this has become clearer to the reader in the revised manuscript.

*6. Why did the observation only do in cartons 21,24, 46,63 and 65? Because we could see that segments’ coverage on cartons 5 and 6 are also over than 90%.*

Thank you for bringing up this point. We have made changes in the paragraph (lines 257-267) that describes passing segments to clear up the wording and correct errors.

*7. The discussion part is more like simple introduction rather than a discussion of result which you have.*

We covered the limitations of this study as well as its future directions in the discussion, but we have also now added some to the discussion surrounding the mutations we were able to identify (lines 372-374).

### Reviewer #6

*The abstract could use a more comprehensive summary of results – e.g. qPCR results, more description of genome coverage (significant correlation with qPCR result?) and/or phylogenetic clustering of resulting sequences.*

We have added to the abstract: "...with higher viral copies corresponding to better sequencing success. The sequences clustered phylogenetically within the rest of the cattle outbreak sequences reported."

*A Spearman correlation should be performed to support the following claim “The method yielded an overall average sequencing depth of 8,176x across all samples, with individual samples varying from 325x to 29,256x. This correlated with the input vRNA amount, with less input vRNA yielding lower depth in sequencing.”*

Thank you for this suggestion. We performed a Spearman's correlation on the aforementioned claim and found that the claim is well-supported. The correlation coefficient was 0.946, with a p-value of 3 x 10^-7^. We have added this result to the manuscript on lines 285-287.

*The quality of the Fig. 2 phylogeny needs to be improved for readability/interpretation. If a tree at higher resolution with less node crowding cannot be made in Auspice, recommend downloading the tree and metadata files from Auspice and re-creating the tree. Several tree tips in Fig. 2 are white/gray – do these not have location metadata? Besides trait inference, did sequenced samples cluster with other specimens from the state? It appears possible for Michigan samples but not Colorado but given the low quality/crowding in the tree is difficult to see. A description of clades that sequenced samples fall into may help.*

We have remade Figure 2 based on your suggestion to reduce vertical crowding of the points. We attempted to generate a new tree in TreeViewer and iTol, but we found that the number of points was not conducive to display in either of these programs. With 1626 points, there must be some node overlap to allow the tree to fit on a single, readable page. With this in mind, we have changed the aspect ratio of Figure 2 to allow the points more room to spread out vertically, which we hope improves the readability of the tree. As far as quality, the figure that was uploaded to PLOS ONE (and that we can download from our submission portal) was of much higher quality than the one included in the pdf manuscript. The loss in quality seems beyond our direct control but we hope the editors can propagate a high resolution version when circulating this revised version for review.

To address the second part of your question, our samples from Michigan fell into a clade composed of 188 sequences from Michigan, Ohio, and Texas. Our samples from Colorado fell into the clade defined by NA:N71S, which includes 1163 sequences from across the country. Cartons 46 and 65 clustered more closely than any others, right next to 3 sequences from the Broad institute's retail milk sampling of Colorado. These sequences are within a larger clade containing sequences from Texas. We have added these sub-trees as supplemental figures S2-S4.

*Further description of sub-consensus mutations could be informative as the samples are pooled from many animals and detection of low-frequency mutations could indicate whether H5N1 was derived from one or many individuals.*

We attempted to perform haplotyping on these samples in order to understand how many infections might be contributing to our sequence. The short amplicons did not contain many mutations that were shared on a single amplicon, making it quite difficult to determine how many infections our samples were drawing from. We have added a sentence to our discussion of this problem in the discussion: "The difficulty of deconvoluting the tiled-amplicon reads is a major downside of our approach compared to whole-segment sequencing." (line 400-402) We also added information about the total number of sub-consensus AA mutations we observed in our passing sequences (line 352-353).

*The caveat that the H5 qPCR standard curve was dsDNA, which is not ideal for RNA virus absolute quantification should be mentioned.*

We have added this caveat to the results on lines 270-271: "In the interest of developing the assay quickly, we used double-stranded DNA as a standard rather than transcribed or viral RNA."

*“resulting in viral titers in milk of at least 107 TCID50 per mL (10).” should be “milk titers up to 107 TCID50 per mL”. Could also cite* [*https://www.nature.com/articles/s41586-024-07849-4*](https://urldefense.com/v3/__https://www.nature.com/articles/s41586-024-07849-4__;!!Mak6IKo!OaACwpYYR_y-vymeqqDyO3IGwSpLiDM7HpS92crmgcyQg4kTyOE5ZRDW0C2uuBfpan25iA6RCJNrhSY$)

Thank you for your comment. The changes have been made on line 58.

*Cited preprint 13 is now published and should be updated, other preprints should be checked for publication status.*

We have done this and updated the requisite papers (see response to Journal Requirements). We were unable to find a published paper corresponding to Nguyen et al. or Lauring et al., so those references have been left as preprints.

*The last paragraph of the introduction contains text more appropriate in the results and discussion sections, consider revising.*

We have revised the last paragraph of the introduction to address this concern (lines 82-96).

*Was the Ct cutoff of 39 based on limit of detection or other assay testing?*

When the news of the cattle outbreak of H5 began spreading, we quickly pivoted to developing an H5-specific assay to detect H5 RNA in retail dairy samples. We wanted to develop this assay as quickly as possible to remain on the front end of the outbreak, and we did not do strict testing to determine a limit of detection or other assay testing to determine a reliable cutoff. We were most interested in prioritizing samples for sequencing. We found that samples with Cts above 39 were much more likely to test positive in one replicate and negative in another, which we also considered to be a negative result. The Ct cutoff was based on our observations, rather than assay testing. We acknowledge that this is sub-optimal and we would have loved to be more rigorous in assay development for this project.

*Table 3 could be converted to a heatmap to improve readability*

We have turned Table 3 into a heatmap (Fig 2) to better demonstrate the differences in coverage.

*Mutation PB2:M631L is common in H5N1 sequences from dairy cattle. Was this mutation not prevalent during the screening time described here? Why this mutation was not observed should be explained.*

This mutation was present in our reference sequence, so it is present in all of our sequences. It does not get flagged as a variant through our pipeline because it is contained in our reference. We have added an explanation on lines 336-338 that we are detecting mutations that are novel relative to the cattle outbreak, rather than focusing on mutations that have already been universally detected in the outbreak and described by others.

*Putative reasons for repeated sampling of rare mutants could be discussed (PB1:171, PA:24). Are the sequences used in “% of nextstrain H5N1 cattle outbreak sequences with mutation” largely from a different source, e.g. could these be mutations associated with milk? A brief description of the referenced nextstrain sequences would be informative : # of sequences, collection dates and locations.*

Interesting point. Cartons 46 and 65 share two mutations that are incredibly rare in other published sequences. This could be because those two cartons were sourced from the same plant code, potentially drawing from the same pool of infected cattle. We investigated the source of each of the other rare mutants in our mutation table. For PB1:171, the three other sequences with this rare mutation are monophyletic with our samples that contain it. (All of them are retail dairy samples, and it is possible that they are from the same plant code as well.) For PA:24, the sequences with this mutation do not cluster together, but they are all sourced from cattle or retail dairy. Each of the two sequences with PA:36T mutations are from cattle, but they do not cluster closely on the tree. For NS:79, the mutation we observed is in a closely related clade with sequences from turkeys, cats, and cattle. As far as we can tell, all instances of MP:28T are in cattle sequences. New figures S2-S4 may also help to answer this question.

*In S3 table, dholab_carton_0028 result is 68.48, is this an error (50 cycles reported in methods)?*

See discussion of this point in response to reviewer #2. Thank you for noticing this mistake.

### Reviewer #7

*It would be highly beneficial to compare the tiled method to universal primer set approach. A comparison would enable future users to choose the most appropriate genome amplification approach.*

We tested the universal primer set approach, but we do not have enough data to enable rigorous comparison of success rates. We made an attempt at sequencing the five samples with the highest vRNA amounts. During the quality control process, only one of these samples amplified as assessed by agarose gel. That one sample was sequenced with some success, but the other samples were not sequenced because the amplification was not sufficient. We do not have a one-to-one comparison of sequencing success. While the universal primer method has many advantages and is likely a better choice for most applications, in this instance we found success using a tiled amplicon approach, since the universal primer approach only worked for a sample with particularly high viral load.

*I suggest a title change to focus on the sequencing method since the sampling was relatively limited and based on convenience (non-systematic). The major strength of the paper is the use of a novel approach to sequencing influenza viruses. However, the title suggest that systematic surveillance was conducted throughout United States whereas retail dairy products were only purchased in Wisconsin.*

We have changed the title to emphasize our amplicon sequencing approach. While we purchased dairy products in Wisconsin, they were produced around the country (see our explanation on lines 109-115). We think this sampling strategy is an important contribution to the paper. We were able to get samples from 20 different states. While these samples do not rise to the level of a systematic sampling, they do capture a good portion of the country, especially states where milk is produced.

*Can you comment on the timings of collection/purchases? Was milk purchased weekly, daily, etc? Was each carton a distinct brand (No need to mention brand/trade names. Please anonymize)?*

We purchased milk weekly or bi-weekly through the period in this study. We aimed for the greatest number of different plant codes, which represent the facility where the milk was processed. We ended up with cartons from 29 different plant codes. We have added this information to the manuscript on lines 101 and 115.

*Please report proportion of samples positive by qRT-PCR since this will be of interest to readers.*

We have added the percentage to line 263, where we report our qRT-PCR results.

*From the genomes sequenced, how does it compare to other isolates from the same state?*

We compared the sequences we generated to samples from different states in Figure 2. Our samples from Michigan fell into the same sub-clade as all known sequences from Michigan. Our samples from Colorado were locally clustered with Texas and Colorado sequences. It is more difficult to compare tree placement relative to known Colorado sequences, since Colorado sequences do not cluster together on the outbreak tree.

*“Because pasteurization neutralizes infectious virus…” It would be more appropriate to use the term “inactivates” instead of “neutralizes” since neutralization of virus commonly refers to the preventing infectious virus to bind to host cells, whereas inactivation is a general term that refers to eliminating the ability of viruses to infect a host.*

Thank you for this suggestion. It has been changed on line 72.

*Data in Table 3 can be made more digestible with a graph*

We have changed Table 3 into a heatmap, now referred to as Fig 2.

*Throughout the paper, please report log copies/mL instead of copies/mL. It is typical for viral RNA titers to be reported in the logarithmic scale because differences in orders of magnitude are more biologically relevant.*

We have made this change.

*What is the rationale for the A/bovine/Texas/24-029328-01/2024? Where is this strain located in the phylogenetic tree? While it is temporally the earliest known isolate, it is not necessarily the most ancestral/closest to the last common ancestor. In other words, the first detection of a virus is not always the first actual spillover of a virus, although it can be. It would be informative to compare your sequences to each representative subclades within the H5N1 isolates from cattle. E.g. A255V may have expanded in the lineage of A/bovine/Texas/24-029328-01/2024 and was not found in the last common ancestor within the clade of cattle H5N1 viruses.*

We recognize the downsides of using this sequence as a reference. However, we selected this reference early in our study and built our workflow around it. The sample has relatively low divergence against the inferred root of the Nextclade tree (0.0017). PB2:255A is the only mutation we found in the reference that is not widely distributed in other cattle sequences. Additionally, the sequence is high quality (only 3 N's at the nucleotide level) and somewhat well known in the field as the earliest known isolate. Ideally, we would compare the mutations to the inferred root as Nextclade does, but since that inferred root is susceptible to change as more sequences are analyzed, we think it is best to use a real isolate. The sequence's relatively low divergence, ease of reference as the first known isolate, and existence as a real sequence (rather than an inferred root) make us feel comfortable using it as a reference in this study.

The following figure shows the location of the reference sequence on Figure 2:

*Please note that there are significant reassortments for clade 2.3.4.4b. Comparisons of mutations are only useful if the segments share the same lineage. Since the cattle outbreak appears to be a result of a single spillover and subsequent farm-to-farm spread, this analysis is appropriate. This may not be the case if reassortments are detected in the phylogenetic analysis.*

With the recent news of two D1.1 spillovers into cattle, the downsides of this approach with respect to recombination are clear. Phylogenetic analysis using concatenated genomes is inadequate when reassortment is in play. Additionally, our tiled amplicon scheme is optimized for B3.13 viruses, creating another obstacle to our strategy moving forward. However, with the focus of this paper being the B3.13 outbreak up until November 2024, we still think these analyses are appropriate within the scope. We have added a paragraph to the "Limitations of this approach" section detailing these concerns (lines 414-416).

*Figure 1. I noticed that there are areas in the genome where coverage drops precipitously. What are possible explanations of this observation? Failure of a primer set to amplify its target?*

That is exactly what is happening. We have added more explanation of this phenomenon to the Figure 1 caption.

*Fig S1. Please use a different color scheme for 2.3.4.4 clades. It is difficult to distinguish a/b/c/d/e/f/g/h*

We have altered this figure to make the colors much more clear.

# References

1.	Wallace HL, Wight J, Baz M, Dowding B, Flamand L, Hobman T, et al. Longitudinal screening of retail milk from Canadian provinces reveals no detections of influenza A virus RNA (April–July 2024): leveraging a newly established pan-Canadian network for responding to emerging viruses. Can J Microbiol. 2025;71: 1–7. doi:10.1139/cjm-2024-0120

2.	Hu X, Saxena A, Magstadt DR, Gauger PC, Burrough ER, Zhang J, et al. Genomic characterization of highly pathogenic avian influenza A H5N1 virus newly emerged in dairy cattle. Emerg Microbes Infect. 2024;13: 2380421. doi:10.1080/22221751.2024.2380421

3.	Spackman E, Jones DR, McCoig AM, Colonius TJ, Goraichuk IV, Suarez DL. Characterization of highly pathogenic avian influenza virus in retail dairy products in the US. J Virol. 2024;0: e00881-24. doi:10.1128/jvi.00881-24

4.	Caserta LC, Frye EA, Butt SL, Laverack M, Nooruzzaman M, Covaleda LM, et al. Spillover of highly pathogenic avian influenza H5N1 virus to dairy cattle. Nature. 2024;634: 669–676. doi:10.1038/s41586-024-07849-4

5.	Jones E, Oliphant T, Peterson P, others. SciPy: Open source scientific tools for Python. 2001. Available: http://www.scipy.org/

6.	Rehman S, Prasetya RR, Rahardjo K, Effendi MH, Rantam FA, Rahmahani J, et al. Whole-genome sequence and genesis of an avian influenza virus H5N1 isolated from a healthy chicken in a live bird market in Indonesia: accumulation of mammalian adaptation markers in avian hosts. PeerJ. 2023;11: e14917. doi:10.7717/peerj.14917

7.	H5N1 Genetic Changes Inventory. Centers for Disease Control and Prevention, National Center for Immunization and Respiratory Diseases; 2012 Jun.

8.	National Milk Testing Strategy | Animal and Plant Health Inspection Service. [cited 14 Mar 2025]. Available: https://www.aphis.usda.gov/livestock-poultry-disease/avian/avian-influenza/hpai-detections/livestock/nmts
