# Supplementary material for: Amplicon sequencing of pasteurized retail dairy enables genomic surveillance of H5N1 avian influenza virus in United States cattle
Source: PLoS One. 2025 Jun 13;20(6):e0325203. doi: 10.1371/journal.pone.0325203 (PMC12165699; doi:10.1371/journal.pone.0325203)
Supplement: Attachment — Submitted filename: response_to_reviewers_auresp_2.docx [file pone.0325203.s013.docx]

Dear Dr. Ayi Vandi Kwaghe,

We would like to thank the editor and reviewers for their helpful comments, which have strengthened the manuscript and allowed us to make it more clear. We are excited to send you the revised manuscript, titled "Amplicon sequencing of pasteurized retail dairy enables genomic surveillance of H5N1 avian influenza virus in United States cattle." We hope that it is now acceptable for publication in *PLOS ONE*.

## Reviewers' Comments:

### Reviewer #1:

*This added section could be more clearly written i.e. ……..Swine are often considered a mixing vessel for recombination between bird, human, and swine flu strains (add reference). This is reflected in the strict biosecurity protocols in use on pig farms. Just like dairy farms, these operations have retail outputs (such as meat ?) which may prove helpful to monitoring these viruses. Performing (asymptomatic?) genomic surveillance (on healthy/asymptomatic animals) at the population level by sampling outputs (meat?) from these locations may (generate data to) help inform risk assessments. However, it is unlikely that this approach (i.e testing milk samples to monitor H5N1 in dairy cattle ?) can be simply reconfigured to swine or another system given the need for quick turnaround and traceable supply chains (please clarify what is meant here ). Dairy ? (Milk ) is pooled between many animals, often sold within a month of production, and contains a marker of where it was produced. It's possible that extending monitoring to other agricultural settings would require orthogonal (please clarify what is meant here) approaches like wastewater or air monitoring..’*

We have edited this section to be more clearly written: “Swine are often considered a mixing vessel for recombination between bird, human, and swine flu strains [51]. This is reflected in the strict biosecurity protocols in use on pig farms. Just like dairy farms, these operations have retail outputs, like meat, which may prove helpful to monitoring these viruses. Performing viral genomic surveillance on asymptomatic animals at the population level by sampling outputs from these locations may generate data to help inform risk assessments. However, it is unlikely that the same approach used for milk samples in this study can simply be reconfigured to swine or another system. There is a need for quick turnaround and traceable supply chains to produce geographically informed, actionable information. Milk is pooled between many animals, often sold within a month of production, and contains a marker of where it was produced. It is possible that extending population level monitoring to other agricultural settings would require alternative approaches like wastewater or air monitoring.”
